# Development, use and evaluation of a national digital platform for physiotherapy during the COVID-19 pandemic - lessons learned

**DOI:** 10.1186/s12913-025-13164-z

**Published:** 2025-08-19

**Authors:** A. Svensson-Raskh, J. Danielsbacka, C. Fridén, M. Fagevik Olsén, M. Nygren-Bonnier

**Affiliations:** 1 Department of Neurobiology, Care Sciences and Society, Division of Physiotherapy, Karolinska Institutet, Stockholm, Sweden; 2 Medical unit Allied health professionals, Women’s Health, and Allied Health Professional Theme, Karolinska university hospital, Stockholm, Sweden; 3 Department of Physical Therapy, Sahlgrenska University Hospital, Gothenburg, Sweden; 4 Division of Physiotherapy, School of Health, Care and Social Welfare, Mälardalen University, Västerås, Eskilstuna, Sweden; 5 Department of Neuroscience and Rehabilitation/Health and rehabilitation, Sahlgrenska Academy, University of Gothenburg, Gothenburg, Sweden

**Keywords:** Knowledge mobilization, COVID-19, Digital platform, Physiotherapy, Descriptive study

## Abstract

**Background:**

During the COVID-19 pandemic, the Swedish healthcare system was challenged, and the need for knowledge regarding how to treat, rehabilitate and evaluate patients was acknowledged from healthcare professionals’ perspective. In March 2020, three clinical researchers constructed a digital platform to facilitate communication among physiotherapists at a national level in Sweden. The purpose of this study is to describe the development process of the Swedish Physiotherapy Platform (SwePPt) and further, to evaluate the need for support and knowledge for physiotherapists in Sweden during the COVID-19 pandemic.

**Methods:**

A descriptive study, containing a process evaluation based on retrospective quantitative and qualitative data from the platform, field notes, memos and a digital survey about the use of the platform.

**Results:**

From April 2020 to January 2023, 26 webinars were held, reaching a minimum of 25 and a maximum of 204 computers. A total of 1854 visitors registered at SwePPt. The visitors represented 20 of 21 regions of Sweden and worked in hospitals, regional or municipal primary health care, private care, and other areas. The survey was answered by 228 participants (86% female, mean 51 (9.2) years) with 98% being physiotherapists. The majority (28.9%), worked in hospitals. Material from the SwePPt was used in clinical everyday life, as reported by 89%, and 61% downloaded materials related to treatment methods (80%), assessment instruments (61%) and articles (50%) and 70% reported they shared the information with others.

**Conclusion:**

The evaluation of the SwePPt discovered that the digital platform was an important source of reliable, up-to-date knowledge and was used to facilitate clinical knowledge mobilization. Furthermore, it is recommended to establish a structured approach for knowledge sharing to be prepared for future crises.

**Trial registration:**

An ethical statement was registered by the Swedish Ethical Review Authority, with Dnr 2023-00040-01.

**Supplementary Information:**

The online version contains supplementary material available at 10.1186/s12913-025-13164-z.

## Background

As the COVID-19 pandemic hit Sweden in early 2020, there was limited knowledge about the management of patient care during a pandemic and how different healthcare groups should organize themselves to best assist patients. The need for knowledge was great across all relevant professional groups, and clinical experiences were shared internationally by countries affected by the pandemic at an early stage [1]. However, there was a lack of structured coordination among relevant professional groups, as well as regionally and locally, regarding collaboration and knowledge mobilization. Therefore, different professions in Sweden handled the pandemic in different ways.

Physiotherapists in Sweden are an important part of the healthcare team and involved in the various parts of the healthcare chain, from emergency and intensive healthcare to rehabilitation in regional and municipal primary healthcare. During the COVID-19 pandemic, the role of physiotherapy became more tangible and even more prominent [2]. For critically ill patients, interventions such as prone positioning, lateral recumbent position, and various forms of breathing exercises became crucial aspects of healthcare. As the patients recovered, physiotherapeutic rehabilitation followed in the form of gradual mobilization, strength training, and activities of daily living (ADL) training when tolerated [3]. For both hospitalized and non-hospitalized patients with residual and persistent symptoms after the acute infection, the need for rehabilitation interventions was significant [4]. As the virus affected multiple organ systems in combination not previously seen with normal seasonal influenza, the need for knowledge, information, and support was extensive, as shown by other studies [5–7].

According to the Swedish Patient Safety Act [8], licensed healthcare professionals and their employer have a shared responsibility to ensure that employees are able to work in accordance with science and proven experience. This was difficult to fulfill at the beginning of the COVID-19 pandemic as evidence and experience was lacking. In May 2020, The Swedish National Board of Health and Welfare published guidelines for healthcare and rehabilitation at the national level, but these guidelines were kept more broad and thus general for all professions [9]. The Public Health Agency of Sweden provided weekly digital updates regarding the spread of the virus and the situation at the acute care settings [10]. Despite the guidelines, physiotherapists perceived an urgent need for increased knowledge. An idea was raised to construct a knowledge platform to facilitate communication among physiotherapists at a national level. The purpose was to share updated materials and experiences related to the clinical management of patients with COVID-19 at all levels of healthcare. A description of how the platform was developed and an evaluation of its content and its significance for users is lacking. Therefore, the aim of this paper is to describe the development process of a national digital platform, the Swedish Physiotherapy Platform (SwePPt) and further, to evaluate the need for support and knowledge for physiotherapists in Sweden during the COVID-19 pandemic.

## Methods

This is a descriptive study containing a process evaluation based on retrospective quantitative and qualitative data.

In March 2020, a discussion was initiated between three clinical researchers (ASR, MFO, MNB), in Sweden, regarding creating a digital platform with the aim of spreading and developing knowledge on a national level concerning physiotherapy for patients affected by COVID-19. The three researchers, the steering group, had solid clinical experience in emergency healthcare covering the areas of respiration, infection, and intensive care. The reasons for developing the SwePPt were a lack of knowledge at all different levels of healthcare, and that many colleagues requested reliable knowledge regarding appropriate treatments, interventions, and outcome measures for patients with COVID-19. The idea was also to develop a knowledge platform that could serve as a liaison between physiotherapists, as well as a source for retrieving material. The work was sanctioned by the Swedish Association of Physiotherapists “Fysioterapeuterna” and its special interest group for cardiorespiratory physiotherapy.

### Context

Sweden is divided into 21 regions and 220 municipalities. The regions have the fundamental responsibility to provide health, medical, and dental care, while the municipalities are responsible for certain areas such as home care services, rehabilitation, and aids for people living in residential care facilities. There are 17,527 registered physiotherapists in Sweden and the majority, 13,851, work in the health care system [11]. The majority of physiotherapists work in the regions at the seven university hospitals, county hospitals, or at primary healthcare centers. About 10% work in the municipalities, providing home care services or working in residential care facilities. There are about 1432 specialist physiotherapists distributed in 17 different specialist areas in Sweden [12]. Intensive care and Respiration are two of the specialist areas that became especially important during the pandemic. However, only a minor group of 20 and 48 physiotherapists respectively, are specialized in those areas [12].

## Methods used to evaluate the platform

### Description of the development

To be able to describe the process and development of the SwePPt, different sources have been used, such as data from the SwePPt, structured field notes and memos from weekly meetings in the steering group.

### Dissemination and use

Information, knowledge about, and the web-link to the SwePPt was spread through the Swedish Association of Physiotherapists [12], the special interest group for cardiopulmonary physiotherapy, the intensive care physiotherapy network and via snowball effect between clinical physiotherapists throughout the country.

Number of digital visits and registered visitors were collected from the digital database “Nationella plattformen för fysioterapeuter om COVID-19” (the National platform about COVID-19 for physiotherapists), at Canvas, https://ki.instructure.com/courses/4193). An estimate of the number of webinars and attendees was collected from the digital video communication platform Zoom (Zoom Video Communications, Inc 2021).

### Evaluation

To quantitively evaluate the use of the SwePPt, an electronic case report form (eCRF) questionnaire was created in the web application REDCap (Research Electronic Data Capture) (Supplementary file 1 and 2). The questionnaire used was developed solely for this study. The content was study-specific and divided into four sections:

1. Collection of demographic data
2. Knowledge of the SwePPt
3. Structure and content of the SwePPt
4. Evaluation of the SwePPt

Most of the questions had fixed answer options, but open-ended questions were also included. Demographic questions included years within the profession, work orientation, type of workplace and if certified as specialist. Respondents’ answers were anonymous.

### Participants in the questionnaire

We aimed to include data from all participants at the SwePPt. Therefore, all visitors who voluntarily registered their email addresses (*n* = 1785) at the SwePPt were sent the electronic case report form (eCRF) questionnaire to evaluate the SwePPt and its use during the fall of 2023. To further disseminate information about the study and recruit participants who had visited the platform but not registered, the steering group shared information about the evaluation of SwePPt, along with a link and QR code to the questionnaire. This was achieved through national physiotherapy meetings, various workplaces, and verbal communication. Those interested in participating registered their email addresses, and a link to the questionnaire was sent to these addresses. A reminder email was sent out two weeks after the initial invitation.

### Statistical analysis

Demographic data regarding the participants were presented as means and standard deviations (SD) for normally distributed continuous variables, as medians and ranges (min– max) for non-normally distributed variables, and as n (%) for categorical data. Statistical analysis was performed using the IBM^®^ SPSS^®^ Statistics software, version 29. The free text sections with reflections by the participants in the survey were analysed manifestly at a low level of interpretation [13] and categorized as presented in Fig. 5. All free-text answers were initially read through several times to create a sense of wholeness, followed by the identification of different topics throughout the text. The different topics were marked as meaning units and then coded into categories and sub-categories. The categories and sub-categories were then discussed and established among all authors to ensure that the material was covered, interpreted, and correctly presented.

## Results

### Description of the development

The SwePPT was built on the digital learning platform Canvas, with permission from Karolinska Institute, Stockholm, Sweden. During March 2020, the steering group collected information on relevant topics, created documents and categorized them into diverse themes, which were then placed in the SwePPt. The first version was launched on the 6th of April 2020. Thereafter, the content continuously expanded with documents produced by the steering group, researchers, and physiotherapists in specialized hospital care, municipal and regional primary healthcare, or through their collaboration. The content was supplemented with guidelines, instructional videos, scientific articles, and relevant news from the media. All visitors to the SwePPt were invited to help build the knowledge base and were encouraged to submit questions and areas of knowledge that needed to be included and covered on the SwePPt and in webinars.

In addition to the material on the SwePPt, weekly webinars commenced 20th of April 2020, via the digital video communication platform Zoom (Zoom Video Communications, Inc 2021), to which everyone was invited. At these digital webinars, various knowledge was presented and summarized, information was given about the care situation regionally and nationally, and various topics were discussed.

The information at the SwePPt was structured in modules with the following themes:

- News.
- Physiotherapy for patients with COVID-19 in Sweden.
- Interventions, evaluation instruments and immersion.
- Physiotherapy management within regional or municipal primary healthcare.
- Recommendations from various hospitals in Sweden.
- e-training.
- Videos with accompanying instructions.
- In the newsfeed.
- For managers and leaders.
- Support for other health care professions.
- References
- Suggested literature.
- Links to web-pages.

### Use of the platform

During the pandemic, 1854 visitors registered at the SwePPt. However, the total number of visitors is unknown as registration was not mandatory. From April to June 2020, weekly webinars (*n* = 10) were arranged. From autumn 2020 to spring September 2021, they were arranged once a month, and then twice per semester. A total of 26 webinars were organized, with the last meeting taking place in January 2023. Approximately 80 computers were connected to the SwePPt during the webinars, of which the minimum number of computers was around 25 and the maximum reach was 204. The exact number of participants is unknown because, in some clinics, several colleagues attended via one computer.

The participants represented all regions of Sweden: 630 (35.4%) worked at hospitals, 516 (29.0%) at regional primary health care, 408 (22.9%) at municipal primary healthcare, 219 (12.3%) at private healthcare, and 81(4.5%) in other areas, including some participants who registered from other countries in Scandinavia and also outside Scandinavia.

The median number of years worked as physiotherapists was 15 years. A total of 56% had worked for more than 20 years as physiotherapists. The most commonly reported specialist degrees were respiration 26%, neurology 24%, and intensive care 13%, see Table 1.

The initial webinars focused on how to handle and treat infected patients in a physiotherapeutic context. This was followed by assessments and treatments of patients during the acute phase followed by the post-infectious phase. Representatives from all over Sweden, from diverse specialties and different professionals, were invited to share their perspective and experiences of managing infectious patients.

### Evaluation of the platform– results from the questionnaire

These results are based on answers from 228 participants who answered the survey. The demographics of the respondents are presented in Table 1.

**Table 1:** Demographics of the participants (*n* = 228) who answered the questionnaire about the use of the digital platform, SwePPt

Age, years (*n* = 200)		
Mean (SD)		51.1 (9.16)
Median (min– max)		52 (30–71)
**Gender**,** female (n**,** %)**		197 (86.4)
**Profession (n**,** %)**		
	Physiotherapist	223 (97.8)
	Other	5 (2.2)
**Professional experience**,** years (n**,** %)**		
	< 2	10 (4.4)
	2–5	7 (3.1)
	6–10	22 (9.6)
	11–20	61 (26.8)
	> 20	128 (56.1)
**Specialist degree (n**,** %)**		
	No	175 (76.8)
	Yes	53 (23.2)
**Specialist fields/degree within the survey group** *(n* **= 53) (n**,**%)**		
	Cardiology	1 (1.9)
	Intensive care	7 (13.2)
	Mental health	1 (1.9)
	Neurology	13 (24.5)
	Obstetrics/Gynecology/Urology	1 (1.9)
	Oncology	2 (3.8)
	OMT	2 (3.8)
	Pediatrics	2 (3.8)
	Primary health care	2 (3.8)
	Respiration	14 (26.4)
	Pain/pain rehabilitation	3 (5.7)
	Geriatric health	5 (9.4)
**Academic degree (n**,** %)**		
	Associate (2-2.5 year)	13 (5.7)
	Bachelor	120 (52.6)
	Master (2 year)	58 (25.4)
	Master (4 year)	17 (7.5)
	Doctoral	20 (8.8)
**Place of work during the pandemic (n**,** %)**		
	Primary health care, municipal	28 (12.3)
	Primary health care, regional	47 (20.6)
	Private establishment	27 (11.8)
	Hospital	66 (28.9)
	Government employee	4 (1.8)
	University hospital	40 (17.5)
	Other	16 (7.0)

*Abbreviations: OMT orthopaedic manual therapy*

Most participants were physiotherapist (98%). The remaining were heads of Physiotherapy departments, in charge of rehabilitation in primary healthcare or occupational therapists. The representation of work placements was broad, from primary healthcare (including home care teams, rehabilitation teams, short term care facilities and residential care homes) to university hospitals and covered many fields of expertise. Twelve out of 17 different physiotherapy specialities in Sweden were represented by the participants in the study, see Table 1. The participants represented 20 out of 21 health care regions in Sweden.

**Fig. 1:**
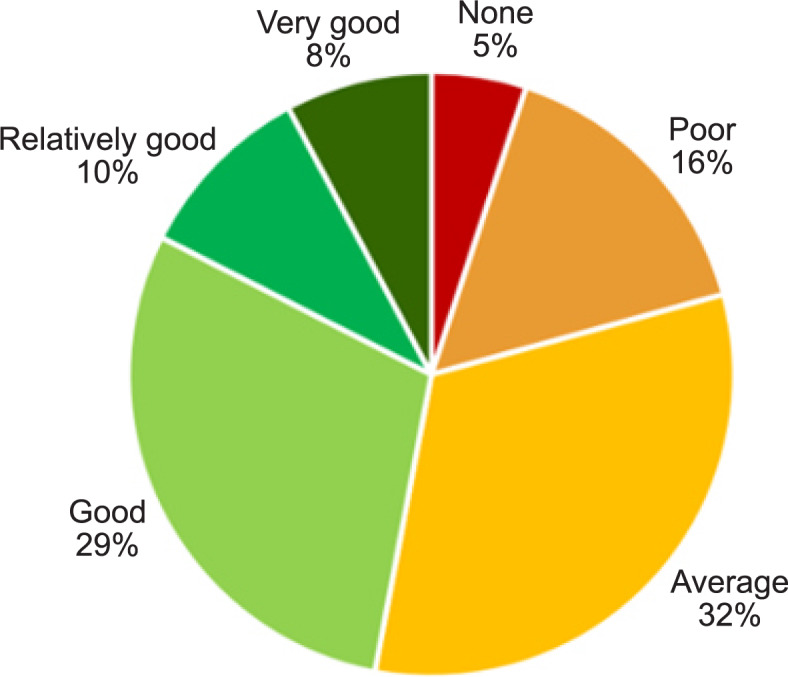
The distribution of knowledge levels regarding physiotherapy in infectious disease

The knowledge levels regarding infectious diseases in general, and knowledge regarding physiotherapy in infectious disease among the participants at the beginning of the COVID-19 pandemic varied, as presented in Fig. 1.

**Fig. 2:**
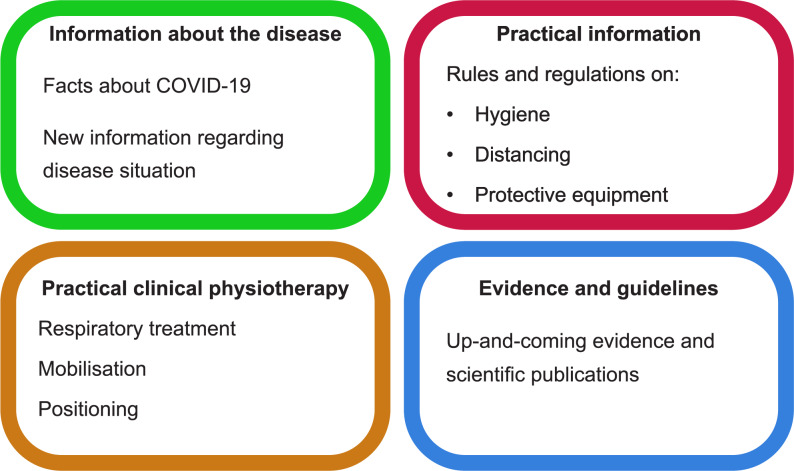
Information given to the participants by their employers at the beginning of the COVID-19 pandemic 2020

32% of participants reported receiving no support from their employer at the start of the pandemic in spring 2020, while 68% reported receiving support/information that consisted of written (83%) as well as verbal (71%) information. The information given could be divided into four different categories; Information about the disease; Practical information; Practical clinical physiotherapy and Evidence and guidelines, see Fig. 2 for details.

Some participants also stated that they received information about the SwePPt from their employers. A large part of the participants (92%) also sought information on assessment, treatment, and intervention from other sources, such as the National Board of Social Affairs and Health.

The participants came in contact with the SwePPt from various sources. A majority, 60%, from the Swedish Association of Physiotherapists or the Cardiorespiratory special interest group. Other channels for receiving knowledge of the SwePPt was through social media, from colleagues or from different networks.

The reasons for logging into the SwePPt were the need for knowledge (85%), the need to build networks (26%), curiosity (25%) and other reasons (4%). Among other reasons the following was expressed; the opportunity to share information from scientific publications, or the experience of a lack of information from one’s own organization, to be able as a manager to follow which areas were important for physiotherapists or in the form of information for personal use in case of personal impact of COVID-19. Almost 82% thought it was easy or very easy to find the link to the SwePPt and a majority of the participants (91.6%) used a computer to access the platform.

**Fig. 3:**
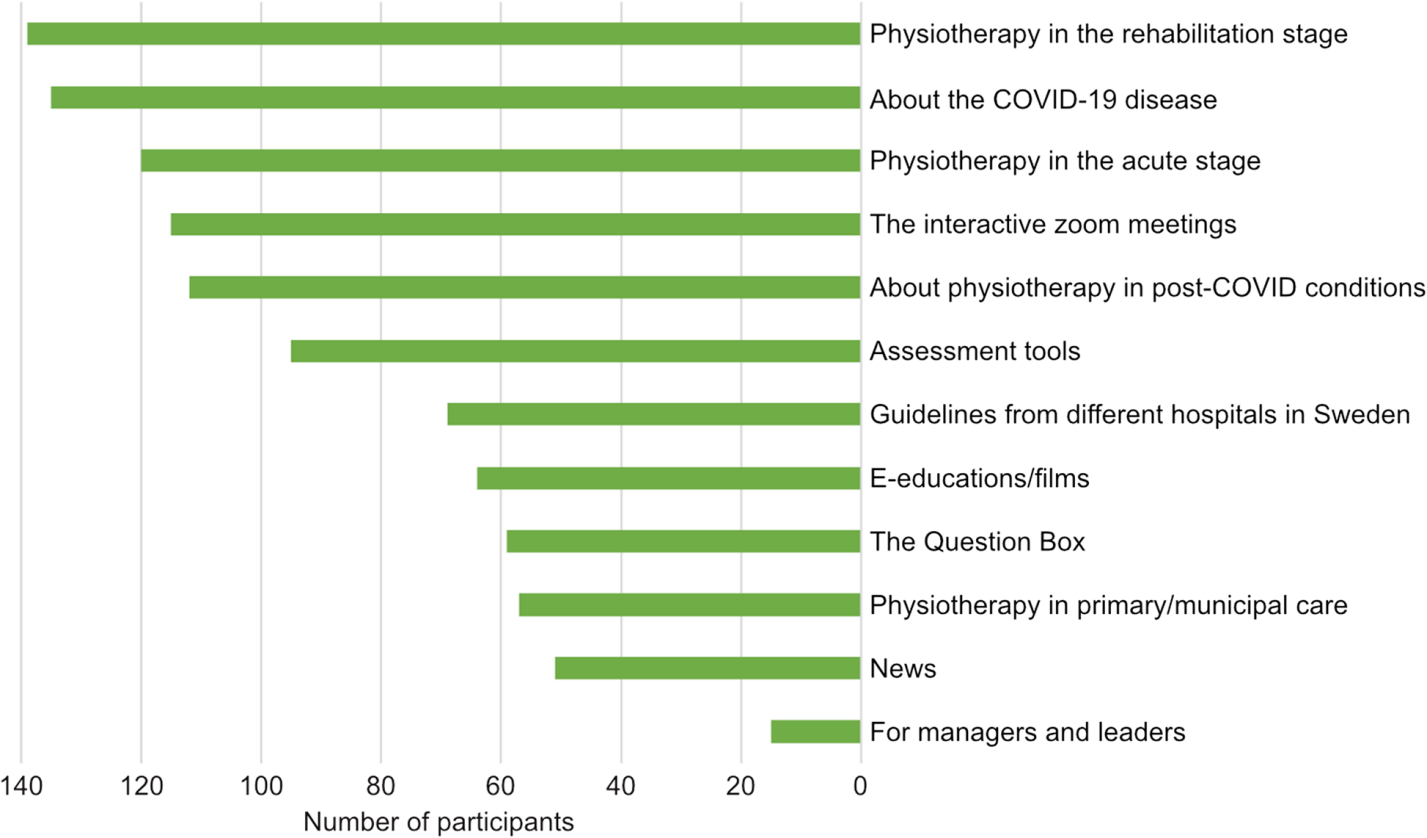
The bar chart presents the participants’ opinions on which parts of SwePPt they considered important. More than one option could be chosen

The results regarding what parts of the SwePPt the participants thought were important to them are shown in Fig. 3.

When asked if the material from the SwePPt came to use in the participants clinical everyday life, 89% stated yes. The use was distributed as: sometimes/average use 33%, great use 50% and very high use 17%. Almost 61% of the participants downloaded materials from the platform. Most downloaded materials regarded treatment methods (80%), assessment instruments (61%) and articles (50%). Other materials downloaded was patient information and educational materials as pictures of positioning. Nearly 70% of the participants shared their downloaded material with others: colleagues 96%, with other members of the hospital health care professionals 40 and 37% with patients.

**Fig. 4:**
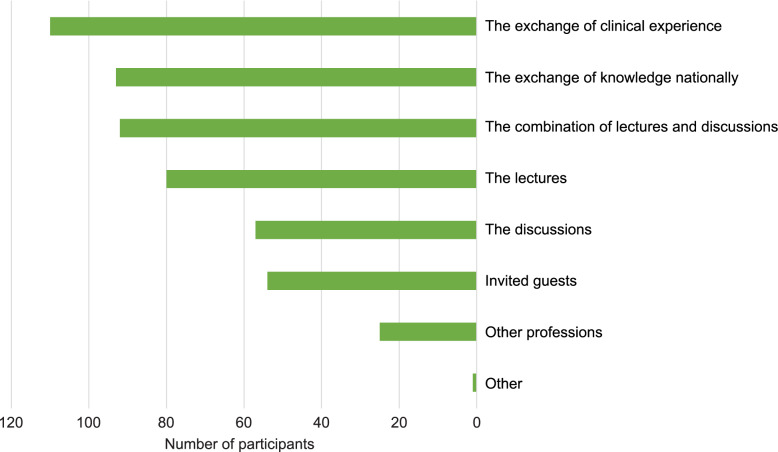
The bar chart presents the participants’ opinions on which parts of the interactive Zoom meetings they considered important. More than one option could be chosen

The interactive Zoom-meetings which was held on the SwePPt was attended to by approximately 68% of the participants, (*n* = 215). Out of these just over a third attended the meetings regularly, nearly a third sometimes, and a third occasionally. When asked about the significance of the interactive Zoom-meetings 19% thought them of little significance, 42% of large significance, and 39% of very large significance, see Fig. 4.

**Fig. 5:**
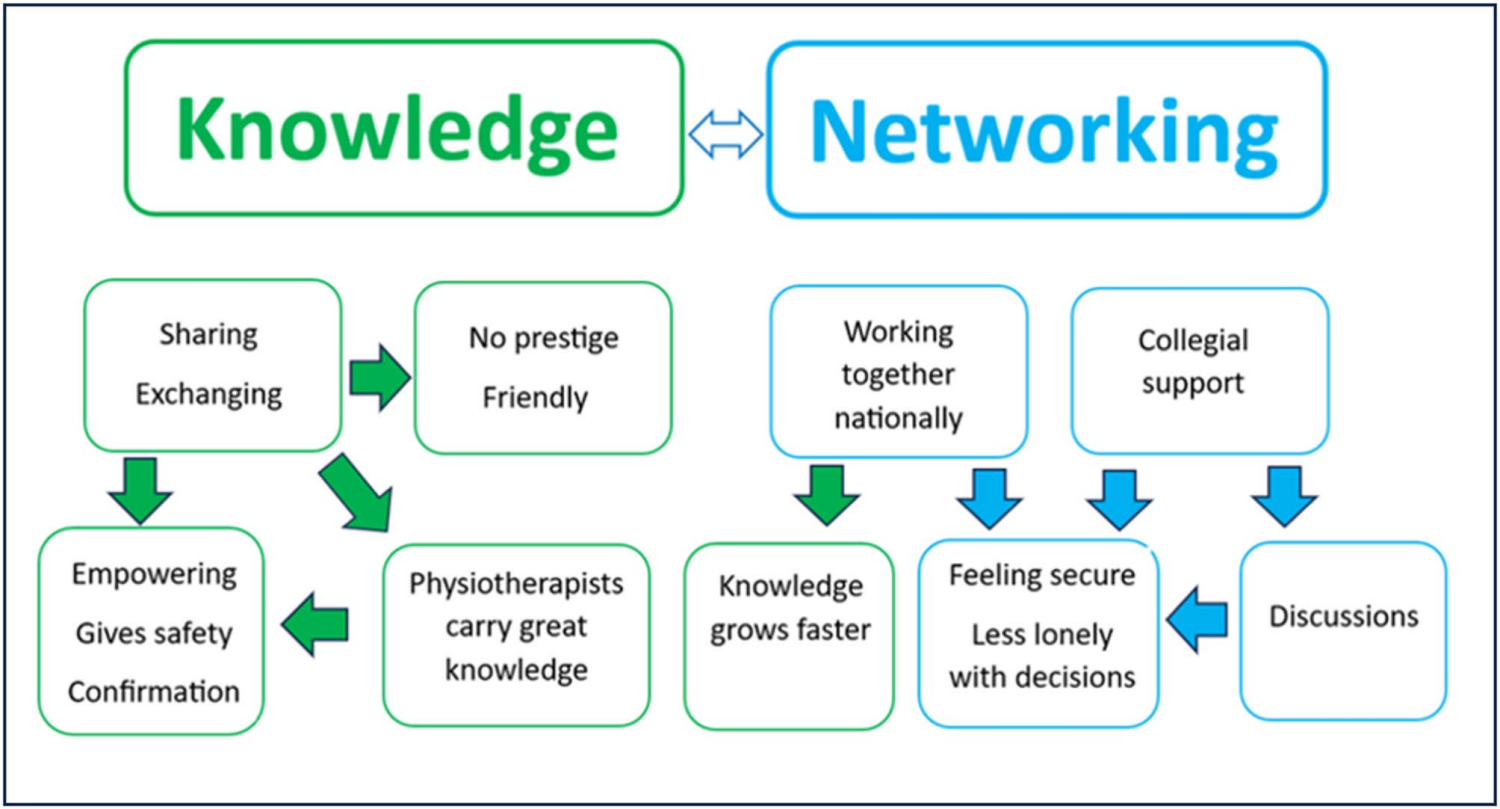
The participants’ experiences of what they retrieved from SwePPt for physiotherapists during the COVID-19 pandemic and beyond. The two main categories identified—Knowledge (green text) and Networking (blue text)—are presented in the figure with corresponding sub-categories

The participants answer to the question on what they retrieved from the SwePPt can be summarized into two main areas– knowledge and networking, see Fig. 5.

It was an advantage that knowledge was concluded on a reliable platform and updated regularly. Moreover, the SwePPt contributed to physiotherapists coming together as a powerful profession, which was expressed as positive. As one participant stated: “We became stronger together during an extremely tough situation”. Additionally, the clinical perspective with opportunities to download materials was valuable to the participants. As one of the participants wrote, “It has been a source of knowledge in several areas, especially in the beginning when not much else was available.” Another participant stated, “A very useful way to reach many people, to share and exchange knowledge, which in this case provided a sense of security.” Regarding networking, one participant stated, “It added valuable knowledge to my everyday clinical life. The platform created security regarding the collective knowledge that existed, was developed, and could be passed on to both colleagues and patients”. The participants also shared thoughts of what they missed on the SwePPt. Aside from some participants having difficulties to log in to the platform or to the interactive Zoom-meetings, thoughts about the platform being hard to navigate was acknowledged. The wish for smaller discussion groups was highlighted since the discussions tended to be too general in the large format. Furthermore, more information about COVID in primary healthcare as well as more post-COVID condition was sought for.

## Discussion

The present study provides insight into how a digital platform can offer nationwide support and contribute to knowledge mobilization among physiotherapists across various clinical fields, in the beginning of a pandemic when little is known, and the need for knowledge is urgent. The platform was developed in parallel with clinical work and through national collaboration. The results of the present study indicate a high degree of knowledge mobilization with a wide outreach nationally.

The development of the SwePPt was rapid and made possible by existing competence, formal and informal networks, and a willingness to contribute to its construction. According to our data, the reach was high, likely because information and meetings were provided digitally. Moreover, the fact that several colleagues could gather around a computer during working hours may have contributed to the scope and exchange of knowledge nationally. Previous studies have emphasized the importance of building knowledge platforms on systems that are easy to understand, intuitive, easy to use and with a high availability, for example easy to log into the system, easy to access information [1, 14–18].

The topics, the webinars, and the invited speakers probably contributed to the engagement in participation and knowledge sharing. Results from the questionnaire regarding the SwePPt presented in this study, show that information from the SwePPt reached 20 out of 21 health care regions in Sweden with a majority of the users being physiotherapists. Two thirds of the participants reported that they did receive support/information from their organization regarding COVID-19 at the beginning of the pandemic. However, the information was mainly practical information focusing on hygiene, distancing, and use of protective equipment, rather than profession-specific issues related to the treatment of patients with COVID-19. The need for clear, reliable, and consistent information, in the beginning and during the pandemic, as well as guidelines, and physically present and responsible leaders has been listed by healthcare professionals as important factors to be able to carry out the work during the pandemic [7, 15, 18–20]. Since there was a vast amount of information about COVID-19 in the media, and it was not uncommon for the information to be unclear and conflicting, it made it difficult for caregivers to interpret the information [15]. The constant influx of new information, policies, and protocols posed significant challenges for organizations and their leaders. The daily task of processing and disseminating this information to staff and clients became overwhelming. This issue was reflected in our survey, where some participants reported that their organizations and leaders struggled to manage the pressure of effectively processing and communicating information to their teams. The participants’ experiences were that in times of distress it is valuable that you have access to reliable information as well as updated information. This made the importance of the information given at the SwePPt even greater since some organizations failed to provide this for their staff. However, participants in this study reported access to knowledge exchange and being able to build a professional network as the two most important areas for accessing information and participating in digital meetings at the SwePPt. To be able to access information useful in clinical work for assessment and treatment, such as downloading materials to oneself, to other health professions or to patients was seen as important according to the participants. Having access to knowledge on an individual professional level, as well as being able to provide information to colleagues, other professions and patients, meant that the knowledge from the SwePPt reached further than just the users of the platform. The need of correct information in times of a great flood of information is acknowledged [14–16, 20–22] and to be able to provide information that has been either developed by researchers and clinical experts gives the information trustworthiness and guarantee a high professional level of information for the own and other professions and for patients.

The information provided through SwePPt was multifaceted and interactive, utilizing various media to disseminate knowledge. This included articles for reading, interactive Zoom meetings featuring expert lectures, as well as clinical discussions and the exchange of clinical experiences and knowledge on a national level. In the survey the participants reported a high appreciation of all these ways to distribute knowledge. In a scoping review on learning it has been shown that physiotherapists prefer learning styles that combine active participation underpinned by clear theoretical concepts [23]. Mixing problem-solving and practical knowledge applications seems to be an optimal learning environment for physiotherapists [23]. By offering a broad supply of different ways to take in and process information and knowledge, the users of the SwePPt could choose their preferred way to process information.

The pandemic highlighted the shortage of specialist trained physiotherapists in Sweden. In some of the most important areas during the pandemic and in its aftermath, there are few specialists (intensive care *n* = 20, respiration *n* = 48, primary health care *n* = 48 and geriatric health care *n* = 57) [12]. These specialists are mainly located in university hospitals and thus have an uneven geographical spread. For example, there are no specialists in intensive care in 15 of 21 regions, which does not provide the conditions for equal health and medical care. Many elderly patients with COVID-19 were cared for at home or at home care services. In the municipalities, responsible for that level of healthcare, there are a total of 14 specialists throughout Sweden [12]. The pandemic highlighted the importance of specialists coming together and having a network to exchange experiences and the willingness from physiotherapists in a clinical setting and researchers to participate at the SwePPt was outstanding.

This study has strengths and limitations. Data regarding the development and use of the platform was retrospectively collected, which can be a limitation due to the risk of missing data and information. In addition, we acknowledge the potential for selection bias in our survey, as our sample of 228 respondents out of 1854 registrants may reflect higher engagement levels among participants. It could be argued that those who responded to the survey were those most in need of support during the pandemic, perhaps those with the most seriously ill patients, those working alone without the opportunity for discussion with colleagues, or those experiencing the most frustration or the least support from employers. This self-selection bias could result in an over-representation of highly engaged users, potentially skewing the results. Despite these limitations, we believe the data accurately reflects physiotherapists’ need for support, provided at the SwePPt, during the pandemic. The questionnaire used was developed solely for this study and thus it was not tested before, which might raise questions regarding its validity and reliability. The responses may be influenced by various biases, such as misunderstanding of questions or social desirability. Thus, the findings have limited generalizability and may not be applicable to a broader population or different samples. However, we still believe that the answers reflect the informants’ experience of the need for support during the pandemic. This is the first study to provide information from a national perspective regarding physiotherapists’ support needs during a pandemic. Whether the information is generalizable outside of a Swedish context is difficult to determine as the environment, needs, context and situation differed in the world during the ongoing pandemic.

In retrospect, using a digital structure, and an existing and established network, made it possible to rapidly design and build a platform with high national availability for clinics during the pandemic. The COVID-19 pandemic has highlighted the urgent need for rapid digital transformation in healthcare. As noted by Torous et al. [17], the pandemic offers an opportunity to accelerate the use of digital technology to provide quality care for individuals with complex health needs. Digital platforms have been essential in overcoming the limitations of physical meetings and ensuring continuous professional development. In Canada, a dedicated website enabled physiotherapists and occupational therapists in Toronto, Ontario to share knowledge regionally [18]. Participants reported that the platform provided timely and relevant information, helping them prepare for COVID-19 patient care and reducing their anxiety about managing COVID-19, concurrent with our findings. The creation of online knowledge hubs has facilitated real-time feedback and iterative user-driven solutions, crucial for rapid design thinking [24]. Establishing virtual communities of practice through social media, webinars, and podcasts further enhances knowledge sharing and professional support. Digital platforms appear to play a crucial role in supporting healthcare professionals during crises [17, 18, 24]. The global trend towards digital knowledge sharing underscores the importance of these tools for healthcare resilience, both in preparing for future crises and for continuous knowledge sharing.

There are some lessons learned from the work with SwePPt regarding crises that affect healthcare. Firstly, the importance of being prepared for future crises with an established infrastructure that can be quickly activated at regional and national level if necessary. Secondly, having pre-prepared platforms that can be quickly activated when needed. Thirdly, to identify stakeholders, organizations, representatives from healthcare organizations and professional associations, universities, and key persons from clinical care. All this should also include collaboration within and between different professional groups for multidisciplinary collaboration and care.

Finally, the results from the evaluation of SwePPt revealed that the digital platform was an important source of reliable knowledge and clinical knowledge mobilization.

## Conclusions

The results from the survey at SwePPt indicate that during an ongoing pandemic, it is important that healthcare professionals receive structured support and an opportunity for knowledge exchange/mobilization. Based on the lessons learned from this study, there is a need to establish platforms and initiate infrastructure that can be activated immediately in acute situations such as pandemics or crises.

## Supplementary Information

Supplementary Material 1

Supplementary Material 2

## Data Availability

No datasets were generated or analysed during the current study.
