# Supplementary material for: Development, use and evaluation of a national digital platform for physiotherapy during the COVID-19 pandemic - lessons learned
Source: BMC Health Serv Res. 2025 Aug 19;25:1108. doi: 10.1186/s12913-025-13164-z (PMC12366103; doi:10.1186/s12913-025-13164-z)
Supplement: Supplementary file 1 — Supplementary Material 1 [file 12913_2025_13164_MOESM1_ESM.docx]

## An English language version of the survey used in present study

**Please complete the survey regarding the evaluation of the national platform.**

**At the start of the COVID-19 pandemic, what was your level of knowledge in:**

**Indicate your level of knowledge in infectious diseases in general at the start of the COVID-19 pandemic:**

0 No knowledge at all

1

2

3 Good knowledge

4

5 Very good knowledge

**Indicate your level of knowledge in physiotherapy for infectious diseases (at the start of the pandemic):**

0 No knowledge at all

1

2

3 Good knowledge

4

5 Very good knowledge

**Did you receive information and support about COVID-19 from your employer during the first wave (spring 2020)?**

Yes

No

**Check the type of information you received from your employer:** Written Oral (Indicate the relevant options)

**Briefly describe the content of the information:**

**Did your employer provide information on appropriate physiotherapeutic interventions to perform for COVID-19 during the first wave (spring 2020)?**

Yes

No

**Check the type of information you received from your employer:** Written Oral (Indicate the relevant options)

**Briefly describe the content of the information:**

**Did you receive information and support about examinations, assessments, and interventions for COVID-19 from other sources such as the National Board of Health and Welfare?**

Yes

No

**Here are some questions about how you came into contact with the national platform for physiotherapists:**

**How did you come into contact with the platform?** (Indicate the relevant options)

Physiotherapy Association Section for Breathing and Circulation (Physiotherapists)

Social media

Colleagues

Networks

Other

**What prompted you to log in to the platform?** (Indicate one or more options)

Curiosity

Need for knowledge/competence

Need for networking

Other Indicate what (short text)

**How easy was it to find the link to the national platform?**

1 Difficult

2

3 Easy

4

5 Very easy

**How did you access the material on the platform (logged in, read posts or information)?**

Phone

Tablet

Computer

Other (Indicate one or more options)

Indicate what (short text)

**Here are questions about the structure/content of the platform:**

**Which parts of the platform were important to you?** Multiple options are possible

Q&A

About the disease COVID-19

Evaluation instruments

Physiotherapy in the acute phase

Physiotherapy in the rehabilitation phase

Physiotherapy for post-COVID

Care in primary care and community

Recommendations from various hospitals in Sweden

E-learning/Films

News

For managers and leaders

References

Interactive Zoom meetings

(Indicate one or more options)

**Did you use the material in your clinical practice?**

Yes

No

**Indicate to what extent you used the material:**

1 Occasionally

2

3 Extensive use

4

5 Very extensive use

**Did you download material from the platform to use in your clinical practice?**

Yes

No

**Indicate the type of material you downloaded from the platform (multiple options possible):**

Articles

Treatment methods

Evaluation instruments

Other

**You indicated that you downloaded other material from the platform - briefly describe what you downloaded:**

(Briefly describe)

**Did you share material posted on the platform?**

Yes

No

**You indicated that you shared material posted on the platform, with whom did you share it?**

Colleague

Other member of the care team

Patient

**Did you participate in the Zoom meetings?**

Yes

No

**How often did you participate in the Zoom meetings?**

Occasionally

A few times

Regularly

**Indicate the significance of the Zoom meetings for you:**

1 Little significance

2

3 Great significance

4

5 Very great significance

**What was important about the Zoom meetings (multiple options are possible)?** (Indicate one or more options)

Lectures

Invited guests

Other professions

Discussions

Clinical experience exchange

Combination of lectures and discussions

National clinical knowledge exchange

Other

**Can you indicate what was important to you about the Zoom meetings (short text)?**

(Indicate in short text)

**What have you taken away from the platform (write briefly)?**

(Write briefly)

**Did you miss anything on the platform?**

Yes

No

**Indicate what you missed on the platform (briefly):**

(Write briefly)

**Question about participation in an interview study:**

**Would you be willing to participate in an interview study about experiences and perceptions of working with patients with COVID-19 and/or post-COVID, as well as your experience of the national platform and need for support and information during the COVID-19 pandemic?**

Yes

No

**Thank you for agreeing to participate in the interview! Please provide your email address in the field below so we can contact you for the interview:**

(Provide your email address in the field here)
