# Supplementary material for: Development, use and evaluation of a national digital platform for physiotherapy during the COVID-19 pandemic - lessons learned
Source: BMC Health Serv Res. 2025 Aug 19;25:1108. doi: 10.1186/s12913-025-13164-z (PMC12366103; doi:10.1186/s12913-025-13164-z)
Supplement: Supplementary file 2 — Supplementary Material 2 [file 12913_2025_13164_MOESM2_ESM.pdf]

Utvärdering av den nationella plattformen

Vänligen, fyll i enkäten som handlar om utvärdering av den nationella plattformen.

Vid Covid-19 pandemins start, vilken var din kunskapsnivå inom:

Ange din kunskapsnivå inom infektionssjukdomar generellt vid Covid-19 pandemins start

◯ 0 Ingen kunskap alls
◯ 1
◯ 2
◯ 3 God kunskap
◯ 4
◯ 5 Mycket god kunskap

Ange din kunskapsnivå när det gäller fysioterapi vid infektionssjukdomar (vid pandemins start)

◯ 0 Ingen kunskap alls
◯ 1
◯ 2
◯ 3 God kunskap
◯ 4
◯ 5 Mycket god kunskap

Fick du information och stöd om Covid-19 från din arbetsgivare under första vågen (våren 2020)?	◯ Yes ◯ No
Kryssa i vilken typ av information du erhöll av din arbetsgivare	☐ Skriftlig ☐ Muntlig<br>(Ange det/de svar som var aktuella)
Ange kort vad informationen innehöll	________________________
Fick du av din arbetsgivare information om lämpliga fysioterapeutiska interventioner att utföra vid Covid-19 under första vågen (våren 2020)?	◯ Yes ◯ No
Kryssa i vilken typ av information du erhöll av din arbetsgivare	☐ Skriftlig ☐ Muntlig<br>(Ange det/de alternativ som var aktuella)
Ange kort vad informationen innehöll	________________________
Tog du del av information och stöd om undersökningar, bedömningar och interventioner vid Covid-19 från annat håll som t.ex Socialstyrelsen?	◯ Yes ◯ No

Här följer några frågor om hur du kom i kontakt med den nationella plattformen för fysioterapeuter

Hur kom du i kontakt med plattformen?
(Ange det/de alternativ som gäller för dig)

- ☐ Fysioterapiförbundet
- ☐ Sektionen för Andning och Cirkulation (Fysioterapeuterna)
- ☐ Sociala medier
- ☐ Kollegor
- ☐ Nätverk
- ☐ Annat

Vad fick dig att logga in på plattformen?
(Ange ett eller flera alternativ)

- ☐ Nyfikenhet
- ☐ Behov av kunskap/kompetens
- ☐ Behov av nätverk
- ☐ Övrigt

Ange vad (kort text)

____
(Ange i kort text)

Hur lätt var det att hitta till länken på den nationella plattformen?

- ◯ 1 Svårt
- ◯ 2
- ◯ 3 Lätt
- ◯ 4
- ◯ 5 Mycket lätt

Hur tog du del av materialet på plattformen (loggade in, läste inlägg eller information)?

☐ Telefon ☐ Platta ☐ Dator
☐ Övrigt
(Ange ett eller flera alternativ)

Ange vad (kort text)

____
(Ange i kort text)

Här följer frågor om struktur/innehåll på plattformen

Vilka delar på plattformen var viktiga för dig?
Flera alternativ är möjliga

- ☐ Frågelådan
- ☐ Om sjukdomen Covid-19
- ☐ Utvärderingsinstrument
- ☐ Fysioterapi i det akuta skedet
- ☐ Om fysioterapi i rehabiliteringsskedet
- ☐ Om fysioterapi vid post-Covid
- ☐ Vård inom primärvård och kommun
- ☐ Rekommendationer från olika sjukhus i Sverige
- ☐ E-utbildningar/Filmer
- ☐ Nyheter
- ☐ För chefer och ledare
- ☐ Referenser
- ☐ De interaktiva Zoom-mötena

(Ange ett eller flera alternativ)

Fick du användning av materialet i din kliniska vardag?

- ◯ Yes
- ◯ No

Ange i vilken omfattning du fick användning av materialet

◯ 1 Ibland/emellanåt
◯ 2
◯ 3 Stor användning
◯ 4
◯ 5 Mycket stor användning

Laddade du ner material ifrån plattformen för att använda i din kliniska vardag? ◯ Yes ◯ No

---

Ange vilken typ av material du laddade ner från plattformen (flera alternativ möjliga)

☐ Artiklar
☐ Behandlingsmetoder
☐ Utvärderingsinstrument
☐ Annat

Du angav att du laddat ner annat från plattformen - skriv kort vad du laddade ner

____
(Skriv kort)

Delade du med dig av material som lades upp på plattformen?

◯ Yes
◯ No

Du har angivit att du delade med dig av material som lades upp på plattformen, till vilka delade du med dig?

☐ Kollega
☐ Annan i vårdteamet
☐ Patient

Deltog du i zoom-mötena

◯ Yes
◯ No

Hur ofta deltog du i zoom-mötena?

◯ Enstaka gånger
◯ Några gånger
◯ Regelbundet

Ange hur stor betydelse zoom-mötena hade för dig

◯ 1 Liten betydelse
◯ 2
◯ 3 Stor betydelse
◯ 4
◯ 5 Mycket stor betydelse

Vad var viktigt med zoom-mötena (flera alternativ är möjliga)?
(Ange ett eller flera alternativ)

☐ Föreläsningarna
☐ Inbjudna gäster
☐ Andra professioner
☐ Diskussioner
☐ Det kliniska erfarenhetsutbytet
☐ Kombinationen av föreläsningar och diskussioner
☐ Det nationella kliniska kunskapsutbytet
☐ Annat

Kan du ange vad det var som var viktigt för dig med zoom-mötena (kort text)

____
(Ange i kort text)

Vad har du tagit med dig från plattformen (skriv kortfattat)?

____
(Skriv kortfattat)

Saknade du något på plattformen

◯ Yes
◯ No

Ange vad det var du saknade på plattformen (kortfattat).

____
(Skriv kortfattat)

Fråga om deltagande i intervjustudie

Kan du tänka dig att delta i en intervjustudie om upplevelser och erfarenheter av att arbeta med patienter med Covid-19 och/eller post-Covid samt din upplevelse av nationella plattformen och behov av stöd och information under Covid-19-pandemin?

◯ Yes
◯ No

Tack för att du vill delta i intervju!
Vänligen ange din e-mail adress i fältet nedan så vi kan komma i kontakt med dig för intervju

____
(Ange din email adress i fältet här)
